# Chaetochromones A and B, Two New Polyketides from the Fungus *Chaetomium* *indicum* (CBS.860.68)

**DOI:** 10.3390/molecules180910944

**Published:** 2013-09-05

**Authors:** Keyang Lu, Yisheng Zhang, Li Li, Xuewei Wang, Gang Ding

**Affiliations:** 1 Key Laboratory of wood science and Technology of State Forestry Administration, Research Institute of Wood Industry, Chinese Academy of forestry, Beijing 100091, China; E-Mail: luky@caf.ac.cn (K.L.); 2 School of Technology, Beijing Forestry University, Beijing 100091, China; 3 Institute of Materia Medica, Chinese Academy of Medical Sciences and Peking Union Medical College, Beijing 100050, China; E-Mail: annaleelin@imm.ac.cn; 4 State Key Laboratory of Systematic Mycology & Lichenology, Institute of Microbiology, Chinese Academy of Sciences, Beijing 100080, China; E-Mail: wangxw@im.ac.cn (X.W.); 5 Key Laboratory of Bioactive Substances and Resources Utilization of Chinese Herbal Medicine, Ministry of Education, Institute of Medicinal Plant Development, Chinese Academy of Medical Sciences and Peking Union Medical College, Beijing 100193, China

**Keywords:** *Chaetomium indicum*, polyketides, chaetochromones, plant pathogens, wood decay

## Abstract

Chaetochromones A (**1**) and B (**2**), two novel polyketides, were isolated from the crude extract of fungus *Chaetomium indicum* (CBS.860.68) together with three known analogues PI-3(**3**), PI-4 (**4**) and SB236050 (**5**). The structures of these compounds were determined by HRESI-MS and NMR experiments. Chaetochromones A (**1**) and B (**2**) are a member of the polyketides family, which might originate from a similar biogenetic pathway as the known compounds PI-3 (**3**), PI-4 (**4**) and SB236050 (**5**). The biological activities of these secondary metabolites were evaluated against eight plant pathogens, including *Alternaria alternata*, *Ilyonectria radicicola*, *Trichoderma viride pers*, *Aspergillus niger*, *Fusarium verticillioide*, *Irpex lacteus* (Fr.), *Poria placenta* (Fr.) Cooke and *Coriolus versicolor* (L.) Quél. Compound **1** displayed moderate inhibitory rate (>60%) against the brown rot fungus *Poria placenta* (Fr.) Cooke, which causes significant wood decay. In addition, the cytotoxic activities against three cancer cell lines A549, MDA-MB-231, PANC-1 were also tested, without any inhibitory activities being detected.

## 1. Introduction

**Figure 1:**
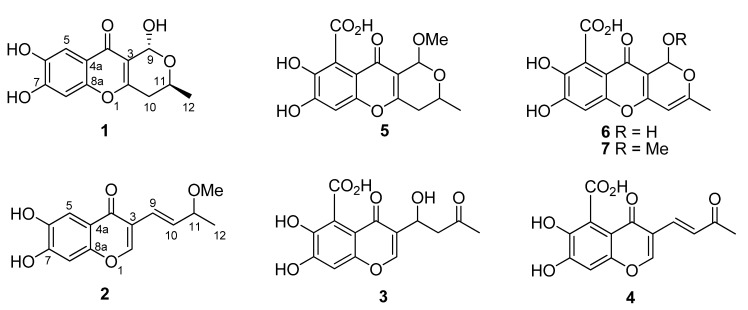
Structures of compounds **1**–**7**.

Fungi of the *Chaetomium* species are the largest genus of saprophytic ascomycetes, which belongs to the *Chaetomiaceae* family. Since Kunze first established this genus in 1817, more than 350 *Chaetomium* species have been described [1]. This fungal spp. is widely distributed in different biotopes, such as soils, marine, animal dung, hair, textiles, plant seeds and some other substrates rich in cellulose. *Chaetomium* spp. are often used to produce cellulose in industry, and are also used as biocontrol agents in agriculture. Due to the diversity of species and of inhabiting-environments, *Chaetomium* spp. might conceive diverse biosynthetic gene clusters, which transform into various secondary metabolites (the fungi languages) to adapt to different ecological environments [2]. Until now more than 200 compounds with a wide range of bioactive effects have been isolated from *Chaetomium* spp., but compared with its richness of species, more bioactive secondary metabolites might be found in this member of fungi [3]. In our ongoing systematic chemical investigation of *Chaetomium* spp. [4,5], two new polyketides, chaetochromones A (**1**) and B (**2**), were isolated from the crude extract of fungus *Chaetomium* *indicum* (CBS.860.68) together with three known analogues PI-3 (**3**) [6], PI-4 (**4**) [6] and SB236050 (**5**) [7] (Figure 1). These secondary metabolites displayed different degrees of inhibitory activity against eight plant pathogens. In this paper, we will present the structure elucidation and bioactive evaluation, and also postulate their plausible biosynthesis. 

## 2. Results and Discussion

**Figure 2:**
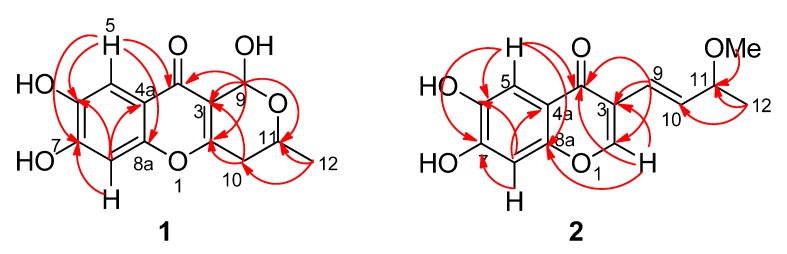
Key HMBC correlations of compound **1** and **2**.

The HRESIMS revealed the molecular formula of chaetochromone A (**1**) as C_13_H_12_O_6_Na (*m/z* 287.0528 [M+Na]^+^; Δ +0.4 mmu) with eight degrees of unsaturation. Analysis of the ^1^H-, ^13^C- and HMQC NMR data for **1** revealed the presence of one methyl, one methylene, two oxygenated methines, eight olefinic carbons (two of which were protonated), one carbonyl group with a chemical shift value of *δ*_C_ 176.6, which implied that this group might be an acetyl group or a ketone one conjugated with two double bonds. Analysis of the NMR data (Table 1), especially HMBC correlations (Figure 2), suggested that a 6,7-dihydroxy-4*H*-chromen-4-one moiety might be present in compound **1**. 

**Table 1:** NMR data for Compounds **1** and **2** in CD_3_OD.

Position	Chaetocromone A (1)		Chaetocromone B (2)	
	*δ*_H_ *^a^* (*J* in Hz)	*δ*_C_ *^b^*, mult.	*δ*_H_ *^c^* (*J* in Hz)	*δ*_C_ *^b^*, mult.
2		165.0	8.15 (s)	153.3
3		117.6		120.9
4		176.6		177.8
4a		117.5		117.9
5	7.39 (s)	108.6	7.37 (s)	108.0
6		146.0		146.3
7		153.2		154.3
8	6.86 (s)	103.7	6.81 (s)	103.6
8a		154.3		155.0
9	5.96 (s)	88.6	6.40 (d, 15.2)	123.4
10a	2.60 (dd, 18.0, 3.6)	35.3	6.41 (overlapped)	134.9
10b	2.68 (dd, 18.0,10.8)			
11	4.50 (m)	63.2	3.82 (m)	79.9
12	1.37 (d, 6.6)	21.2	1.23 (d, 6.6)	21.6
-OMe				56.3

*^a^* Recorded at 500 MHz. *^b^* Recorded at 125 MHz. *^c^* Recorded at 600 MHz. *^d^* Recorded at 150 MHz.

The HMBC correlations from CH_3_-12 to C-10 and C-11 confirmed that C-12 and C-10 were connected with C-11. The C-2/C-3 double bond connection with C-10 was confirmed by the correlation of H-11 with C-2, and H-10 with C-2 and C-3. The correlations from H-9 to C-2, C-3, C-4 and C-11 established one 2-methyl-3,6-dihydro-2*H*-pyran fragment with one keto group anchored at C-3. In the HMBC spectra, the aromatic protons H-5 had correlations with C-4, C-6, C-7 and C-8a, and H-8 had correlations with C-4a, C-6 and C-7, together accounting for the above-mentioned fragments, and comparison with its analogues SB236050 (**5**) [7] and **6** [8], confirmed that **1** contained a 6,7-dihydroxy-4*H*-chromen-4-one moiety. According to the chemical shift values and molecular formula, C-6, C-7 and C-9 each must be connected with one free hydroxyl group, respectively, which finally established the structure of **1** as shown in Figure 1.

The relative configuration was established by analysis of coupling constant and NOESY correlations. The *J* value of H-10b/H-11 (10.8 Hz) implied those two protons H-10b and H-11 to be puseudo-axially. The correlations between CH_3_-12 or H-11 with H-9 did not be observed in the NOESY spectra, which could not determined the relative configuration. We tried to use a modified Mosher’s reaction to determine the absolute configuration of secondary hydroxyl group (9-OH), while the (*R*)-MTPACl/ (*S*)-MTPACl did not react with the 9-OH in **1**, implying that the hydroxyl group (9-OH) might form an intra-molecular hydrogen bond with C-4 to preclude the reaction from happening.

**Figure 3:**
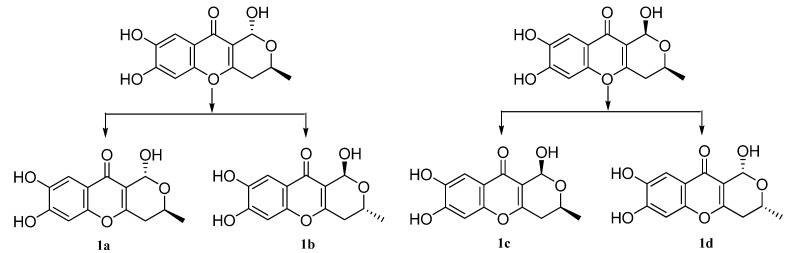
Two possible relative and four absolute configurations of **1**.

**Figure 4:**
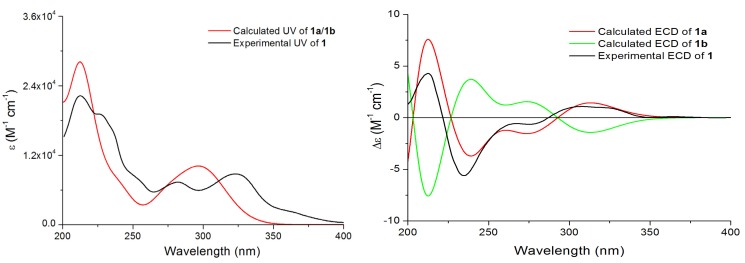
Experimental UV and ECD spectra of **1**, and calculated and ECD spectra **1a** and **1b**.

In theory, there exist two relative configurations and four absolute configurations for compound **1**, as depicted in Figure 3. Conformational analysis combined with NMR data revealed that compound **1** should possess the absolute configuration indicated as **1a** or **1b**. Then the absolute configurations at C-9 and C-11 of **1** were assigned by comparison of the experimental ECD data and the theoretical ECD spectra predicted using time-dependent density functional theory (TDDFT). Since the relative configuration of **1** was established by a NMR method, a pair of enantiomers (**1a** and **1b**, Figure 4) was proposed as the model compounds. The predicted ECD spectrum of **1a** was in good agreement of the experimental ECD spectrum of compound **1**. Thus, the absolute configurations of 9 and 12 were determined as 9*R*, and 11*S*.

The molecular formula of **2** was determined as C_14_H_14_O_5_Na (*m/z* 285.0735 [M+Na]^+^; Δ +0.4 mmu) by HRESIMS data with eight degrees of unsaturation. Compared with NMR data with those of **1**, it revealed that compound **2** had one more double bond, suggesting that **2** possessed one less ring system than **1**. 

**Figure 5:**
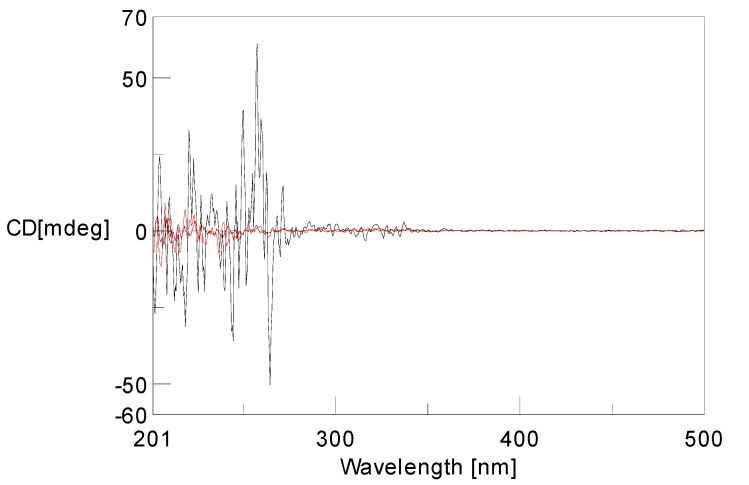
Experimental ECD spectra of **2**.

The NMR data (Table 1), especially the HMBC spectra revealed that compound **2** also possessed the same 6,7-dihydroxy-4*H*-chromen-4-one moiety as that of **1** (Figure 2). The HMBC correlations from H-9 to C-2, C-3 and C-4, H-10 to C-3, CH_3_-12 to C-10 and C-11, and also from OCH_3_-11 to C-11 confirmed that one 4-methoxypent-1-ene fragment was connected with C-3. Thus the structure for compound **2** was characterized. The low specific optical rotation and the lack of significant Cotton effects in the ECD spectrum indicated that **2** comprised a racemic mixture (Figure 5).

The known compounds were determined to be PI-3 (**3**) [6], PI-4 (**4**) [6] and SB236050 (**5**), respectively [7], by comparison of MS and NMR with those reported in the literature. 

**Scheme 1:**
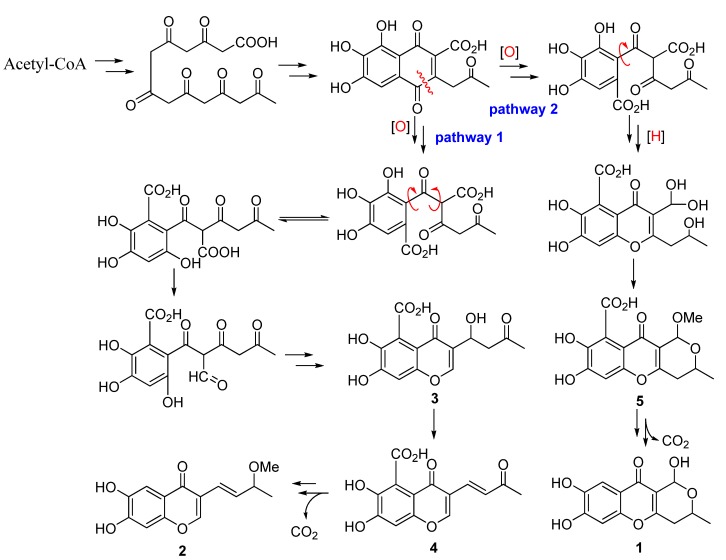
Putative Biosynthetic Pathway for Compounds **1**–**7**.

Chaetochromone A (**1**) possesses the same 7,8-dihydroxy-3,4-dihydropyrano [4,3-*b*] chromen-10 (1*H*)-one core skeleton as SB236050 (**5**) and chaetocyclinone A (**6**), whereas **1** does not have the carboxyl group at C-7 present in its analogues (compounds **5** and **6**), which is the first report of such an arrangement in its congeners. Chaetochromone B (**2**) contains a similar carbon skeleton as PI-3 (**3**) and PI-4 (**4**) except for the absence of the 7-carboxy group. In addition, the aliphatic chain at C-3 between these compounds is also different. Zeek *et al.* investigated the biosynthesis of chaetocyclinone A (**6**) by feeding ^13^C-labelled acetate [8]. The biosynthetic experiment revealed that chaetocyclinone A (**6**) originated from seven labeled acetate units via condensation, reduction, oxidative ring cleavage and other reactions to form the final polyketide product. From the structural features of **2** and **5**, it implies that these two compounds might have the same polyketide origin as that of chaetocyclinone A (**6**) via possible biogenetic pathway 2, while compounds **1**, **3**, and **4** might be biosynthesized from pathway 1 to form the aliphatic chain (Scheme 1). 

SB238569 (**7**) exhibited strong bioactivities against *Bacillus cereus* II, *Pseudomonas aeruginosa* IMP-1, and *Bacteroides fragilis* CfiA metallo-β-lactamses, with K*_i_* values of 79, 17, and 3.4 μM, whereas compound **5** did not display any bioactivities [7]. The only difference between **5** and **7** is the additional double bond at C-11 and C-12, suggesting that the double bond might be the bioactive group. In addition, the presence of a double bond in **7** could change the planar structure of ring C, which might influence the orientation of the C-9 and C-11 substituents leading to the different observed bioactivities between compounds **5** and **7**.

There is not report of bioactivity about compounds **3** and **4**. Compounds **1**–**5** were evaluated the biological activities against eight pathogens including *Alternaria alternata*, *Ilyonectria radicicola*, *Trichoderma viride pers*, *Aspergillus niger*, *Fusarium verticillioide*, *Irpex lacteus* (Fr.), *Poria placenta* (Fr.) Cooke, and *Coriolus versicolor* (L.) Quél (Table 2). Compound **1** displayed moderate inhibitory activity (>60%) against *Poria placenta* (Fr.) Cooke. *Poria placenta* (Fr.) *Cooke*, as one of the important brown rot fungi, rapidly depolymerizes the cellulose in wood without significant lignin removal, which will lead to the destructive decay of wood in buildings and other structures [9]. Our results implied that this fungus might be a potential bio-control agent in wood protection against the brown rot fungi *Poria placenta* (Fr.) *Cooke*. In addition, compounds **1**–**5** was evaluated for cytotoxic activities against three cancer cell lines, including A549, MDA-MB-231, PANC-1 with no strong bioactivities (>40 μm).

**Table 2:** Cell growth inhibitory rate (%) of several plant pathogenic fungi evaluated by compounds **1**–**5** at 200 μg/mL.

Pathogens	1	2	3	4	5
*A. alternata*	41.40	45.60	47.54	51.07	20.61
*I* *.* *radicicola*	31.38	33.92	57.31	5.75	45.32
*T. viride pers*	38.27	36.87	55.41	49.70	48.28
*A. niger*	–	–	59.95	–	–
*F. verticillioide*	23.31	7.10	19.44	33.77	31.98
*I. lacteus* (Fr.)	–	11.76	19.61	22.29	42.17
*P. placenta* (Fr.) Cooke	63.70	20.94	49.48	23.97	20.52
*C. versicolor* (L.) Quél	14.23	8.32	22.88	15.72	21.80

## 3. Experimental

### 3.1. General

NMR spectra were measured on a Bruker AM 600 NMR spectrometer as the internal reference and chemical shifts are expressed in ^TM^ (ppm). TOF-ESI-MS spectra were measured on a Waters Synapt G2 mass spectrophotometer. IR spectra were recorded on a Shimadzu FTIR-8400S spectrophotometer. UV spectra were run on a Shimadzu UV-2550 UV_vis spectrophotometer. Purification was performed by Semi-prep-HPLC with a Lumtech apparatus equipped with UV detector under ODS column (250 × 10 mm; YMC Co., Ltd.). TLC was performed on silica gel GF254 (10–40 *μ*m; Qingdao Marine Chemical, Inc., Qingdao, China). Column chromatography was performed on silica gel (100–200 or 200–300 mesh; Qingdao Marine Chemical, Inc.).

### 3.2. Fungal Material

The fungal strain was identified by one of the authors (Wang, X.W.), and deposited in the lab of Wang X.W. at the Institute of Microbiology, Chinese Academy of Sciences, Beijing. The fungal strain was cultured on slants of potato dextrose agar (PDA) at 25 °C for 10 days. The agar plugs were used to inoculate 250 mL Erlenmeyer flasks, each containing 40 mL of media (0.4% glucose, 1% malt extract, and 0.4% yeast extract), and the final pH of the media was adjusted to 6.5 before sterilization. Flask cultures were incubated at 25 °C on a rotary shaker at 170 rpm for five days. Fermentation was carried out in Fernbach flasks (500 mL) each containing 80 g of rice. Spore inoculum was prepared by suspension in sterile, distilled H_2_O to give a final spore/cell suspension of 1 × 10^6^/mL. Distilled H_2_O (100 mL) was added to each flask, and the contents were soaked overnight before autoclaving at 15 lb/in^−2^ for 30 min. After cooling to room temperature, each flask was inoculated with 5.0 mL of the spore inoculum and incubated at 25 °C for 40 days. 

### 3.3. Extraction and Isolation

The fermented material was extracted with ethyl acetate (5 L, four times). The solution was concentrated to dryness under vacuum to afford a crude extract (10.0 g), which was fractionated by silica gel column chromatography (CC, 10 × 100 cm) using CH_2_Cl_2_–MeOH gradient elution. The fraction (236 mg) eluted with 99:1 CH_2_Cl_2_–MeOH was separated over RP-HPLC (Lumtech; YMC-Pack ODS-A column; 10μm; 250 × 10 mm; 2 mL/min, 0–5 min, 32% MeOH in H_2_O, 5–95 min, 32%–45% MeOH in H_2_O) to afford **3** (5.0mg, *t*_R_ 23.7 min) and **4** (1.8 mg, *t*_R_ 35.4 min). The fraction (660 mg eluted with 98:2 CH_2_Cl_2_–MeOH) was chromatographed on Sephadex LH-20, and then separated by RP-HPLC (Lumtech; YMC-Pack ODS-A column; 10μm; 250 × 10 mm; 2 mL/min, 0–5 min, 25% MeOH in H_2_O, 5–65 min, 25%–55% MeOH in H_2_O) to afford **1** (2.0mg, *t*_R_ 24.2 min) and **2** (1.8 mg, *t*_R_ 30.0 min). The fraction (235 mg eluted with 99:1 CH_2_Cl_2_–MeOH) was chromatographed on Sephadex LH-20, and then separated by RP-HPLC (Lumtech; YMC-Pack ODS-A column; 10 μm; 250 × 10 mm; 2 mL/min, 0–5 min, 30% MeOH in H_2_O, 5–25 min, 30%–100% MeOH in H_2_O) to afford **5** (3.0 mg, *t*_R_ 25.0 min). 

### 3.4. Bioactivity Assay

The bioactive assay experiment of pathogenic microbes followed reference [10], and the cytotoxic tests followed reference [11].

### 3.5. Spectral Data

*Chaetocromone A* (**1**) Brown oil; [*α*]_D_ = −8.0 (*c* 0.05, CH_3_OH); UV (MeOH) *λ*_max_ (log ε) 280.5 nm; IR (KBr) *ν*_max_ 3165 (OH), 1687 (C=O), 1434, 1243 cm^−1^; TOF-ESI-MS *m*/*z* 287.0528 [M + Na]^+^ (calcd 287.0532 for C_1__3_H_12_O_6_Na); ^1^H and ^13^C-NMR data: see Table 1.

*Chaetocromone B* (**2**) Brown oil; [*α*]_D_ = −7.5 (*c* 0.08, CH_3_OH); UV (MeOH) *λ*_max_ (log ε) 286 nm; IR (KBr) *ν*_max_ 3167 (OH), 2956, 1699 (C=O), 1434, 1392, 1170 cm^−1^; TOF-ESI-MS *m*/*z* 285.0735 [M + Na]^+^ (calcd 285.0739 for C_1__4_H_14_O_5_Na); ^1^H and ^13^C-NMR data: see Table 1.

## 4. Conclusions

Diverse secondary metabolites with different complex structure features are isolated from *Chaetomium* spp. Our previous chemical investigation of the endophytic fungus *Chaetomium globosum* led to isolated two classes of secondary metabolites, which included the cytochalasans and azaphilones [3,4]. The former (cytochalasans) displayed strong activities against the human nasopharyngeal epidermoid tumor KB cell line [3], whereas the latter (azaphilones) exhibited activity against the human breast cancer cell line MCF-7 and colon cancer cell line SW1116 with IC_50_ values of 26.8 and 35.4 mg mL^−^^1^[4], respectively. In this report, we reported the isolation from *Chaetomium* *indicum* (CBS.860.68) of two new polyketides, chaetochromones A (**1**) and B (**2**), along with the three known analogues PI-3 (**3**), PI-4 (**4**), and SB236050 (**5**). Compounds **1**–**5** were evaluated the inhibitory activities against several pathogenic microbes, and compound **1** exhibited moderate inhibitory rate (>60%) against *Poria placenta* (Fr.) Cooke., a very important brown rot fungus leading to the destructive decay of wood in buildings and other structures. Compounds **1**–**5** were also tested for cytotoxic activity against three cancer cell lines, A549, MDA-MB-231, PANC-1 with no strong biological effects. Chaetochromones A (**1**) and B (**2**) are a new member of polyketides different from other analogues, which implied that a systematic chemical investigation of *Chaetomium* spp. could provide more novel/new secondary metabolites with a wide range of bioactivities.
